# Femtosecond laser treatment promotes the surface bioactivity and bone ingrowth of Ti_6_Al_4_V bone scaffolds

**DOI:** 10.3389/fbioe.2022.962483

**Published:** 2022-09-23

**Authors:** Su Wang, Miao Zhang, Linlin Liu, Rongwei Xu, Zhili Huang, Zhang’ao Shi, Juncai Liu, Zhong Li, Xiaohong Li, Peng Hao, Yongqiang Hao

**Affiliations:** ^1^ School of Mechanical Engineering, Sichuan University, Chengdu, China; ^2^ Department of Orthopaedics, The Affiliated Hospital of Southwest Medical University, Sichuan Provincial Laboratory of Orthopedics Engineering, Luzhou, China; ^3^ School of Science, Southwest University of Science and Technology, Mianyang, China;; ^4^ Sichuan Provincial People’s Hospital, Chengdu, China; ^5^ Department of Orthopedics Surgery, Ninth People’s Hospital, Shanghai Jiaotong University School of Medicine, Shanghai, China

**Keywords:** femtosecond laser, micro-nano surface morphology, super hydrophilic structure, surface bioactivity, bone tissue growth

## Abstract

In this study, a femtosecond laser with a wavelength of 800 nm was used to modify the surface of a titanium alloy bone scaffold created *via* selective laser melting (SLM). The outcomes demonstrated that the surface morphology of the bone scaffold after femtosecond laser treatment was micro-nano morphology. The hydrophobic structure of the scaffold was changed into a super-hydrophilic structure, improving the surface roughness, which was highly helpful for osteoblast adhesion and differentiation. The femtosecond laser surface treatment *in vitro* samples produced a thick layer of hydroxyapatite (HAP) with improved surface bioactivity. The effectiveness of osseointegration and interstitial growth of the specimens treated with the femtosecond laser surface were found to be better when bone scaffolds were implanted into the epiphysis of the tibia of rabbits. As a result, femtosecond laser therapy dramatically enhanced the surface activity of bone scaffolds and their capacity to integrate with the surrounding bone tissues, serving as a trustworthy benchmark for future biological scaffold research.

## Introduction

Due to its superior mechanical qualities, corrosion resistance, and biocompatibility, titanium (Ti) and its alloys have been the most popular choice of implant materials. In the additive manufacturing process known as selective laser melting (SLM), powder is melted and stacked one layer at a time into complicated three-dimensional parts. In the biomedical arena, it is unquestionably appealing for the tailored preparation of orthopedic implants. At this time, clinical tests using porous titanium alloy scaffolds made by SLM have been successful (Van Hooreweder et al., 2017; Wang et al., 2019; Zhao X. et al., 2020).

Bone scaffolds with strong biocompatibility are implanted into the defected area in bone tissue engineering to give growing space for cells and growth factors and encourage bone tissue regeneration. This approach is seen to be a promising one for repairing bone defects (Turnbull et al., 2018; Wubneh et al., 2018). The failure rate of stent implantation has increased significantly as a result of the inadequate connection between the scaffold and the bone in the initial stage of implantation as it is not favorable for the growth of bone tissue into the scaffold (Gnilitskyi et al., 2019). The micro–nano morphology, roughness, hydrophilicity, and bioactivity of the scaffold surface, which are significant elements determining the effectiveness of osseointegration, have been demonstrated in pertinent research to be able to encourage the formation of bone tissue (Luo et al., 2014; Raimbault et al., 2016). To increase the effectiveness of osseointegration between bone tissue and Ti implants, surface modification is crucial.

With the development of surface modification technology, the osseointegration of different surface modification methods has attracted extensive attention of researchers. Many surface modification methods for bone scaffolds have been reported in previous studies, including chemical methods such as alkali heat, acid etching, and oxidation (Amin Yavari et al., 2015; Pylypchuk et al., 2015; Tan et al., 2016), and physical methods such as grinding, sandblasting, and roughening (Le Guéhennec et al., 2007; Coelho et al., 2009; Abdel-Haq et al., 2011). Fanny (Hilario et al., 2017) synthesized TiO_2_ nanotubes on the surface of a titanium plate by anodic oxidation to obtain better corrosion resistance and biological activity. Wang and Xiong (2018) prepared novel composite coatings using two different surface modification technologies (micro-arc oxidation and grafting hydrophilic polymers), demonstrating better hydrophilicity and abrasive resistance.

Dingyun Zhao et al. (2020) quickly prepared the sodium titanate rutile TiO_2_ bioactive structure on the titanium surface by induction heating, acid etching, and alkali heat. This structure had good adhesion, corrosion resistance, and a strong ability to induce the formation of hydroxyapatite (HAP) in a simulated body fluid, and the formed HAP had excellent long-term stability. Calcium and phosphate, which are necessary for bone formation, are found in HAP, a biocompatible and osteoconduction bioactive ceramic Ca_10_(PO_4_)_6_(OH)_2_. It deposits on the surface of Ti, which is conducive to combining with living bone and promoting the growth of osseous tissue. Kostelac et al. (2022) prepared a 2-grade titanium alloy bioactive coating with HAP as the main component by the plasma electrolytic oxidation process. The cell adhesion biological test showed that the coating had excellent cytocompatibility with human cells, and the cell adhesion performance was improved compared with that of untreated samples. The aforementioned surface modification methods can improve the bioactivity of bone scaffolds. However, problems such as the coating adhesion being so poor that it is easy to peel, chemical pollution, and difficult control of the surface structure are difficult to ignore.

Because laser processing is non-contact, highly repeatable, and produces very little pollution, it is a dependable technique for surface modification (Cunha et al., 2015). Compared to nanosecond and picosecond pulse lasers, femtosecond lasers can prevent thermal diffusion and cause less thermal damage to nearby materials (Lu et al., 2018). Furthermore, by concentrating a femtosecond laser at micro-level spots, the etching trajectory and surface morphology can be accurately controlled. Based on the aforementioned benefits, femtosecond laser technology can stand out from other surface modification techniques and take the top spot among them.

In recent years, we have seen an increase in studies on the surface bioactivity of titanium and its alloys by femtosecond laser therapy, with the aim of directing the femtosecond laser ablation on the surface of titanium alloys to generate micro–nano morphology. The multiscale shape of micro/nano combinations accelerated the development of osteoblasts, according to Tsukanaka et al. (2016). In order to give the titanium surface good hydrophilicity, which is more beneficial for the deposition of HAP and accelerates the adhesion and differentiation of osteoblasts, Lin et al. (2020) conducted a series of regular lattice microstructures on the titanium surface using the femtosecond laser micro–nano-processing technology. Bouet et al. (2019) used a femtosecond laser to change the surface morphology of the Ti alloy which is formed by SLM technology. The results showed that the stent surface modified by the femtosecond laser had more spikes; the surface with more spikes had better hydrophilic performance, stronger bone integration ability, and better antibacterial effect. Although some researchers have studied how to increase the biological activity by applying a femtosecond laser to the surface of Ti/Ti alloy slices, there are few reports on how to increase the biological activity of a Ti alloy bone scaffold and then watch bone tissue grow after implantation.

In this study, the SLM-produced Ti_6_Al_4_V bone scaffolds were directly ablated using the femtosecond laser micro–nano-processing technology. Surface morphology, roughness, and hydrophilicity define the surface properties of the scaffolds. To determine the promoting impact of femtosecond laser surface treatment on the surface activity and osteogenic growth ability of the bone scaffold and to obtain a bone scaffold with better osseointegration ability in the early stage of implantation, the *in vitro* activity and rabbit tibial implantation experiment were carried out.

## Experiment

### Preparation of samples

**FIGURE 1:**
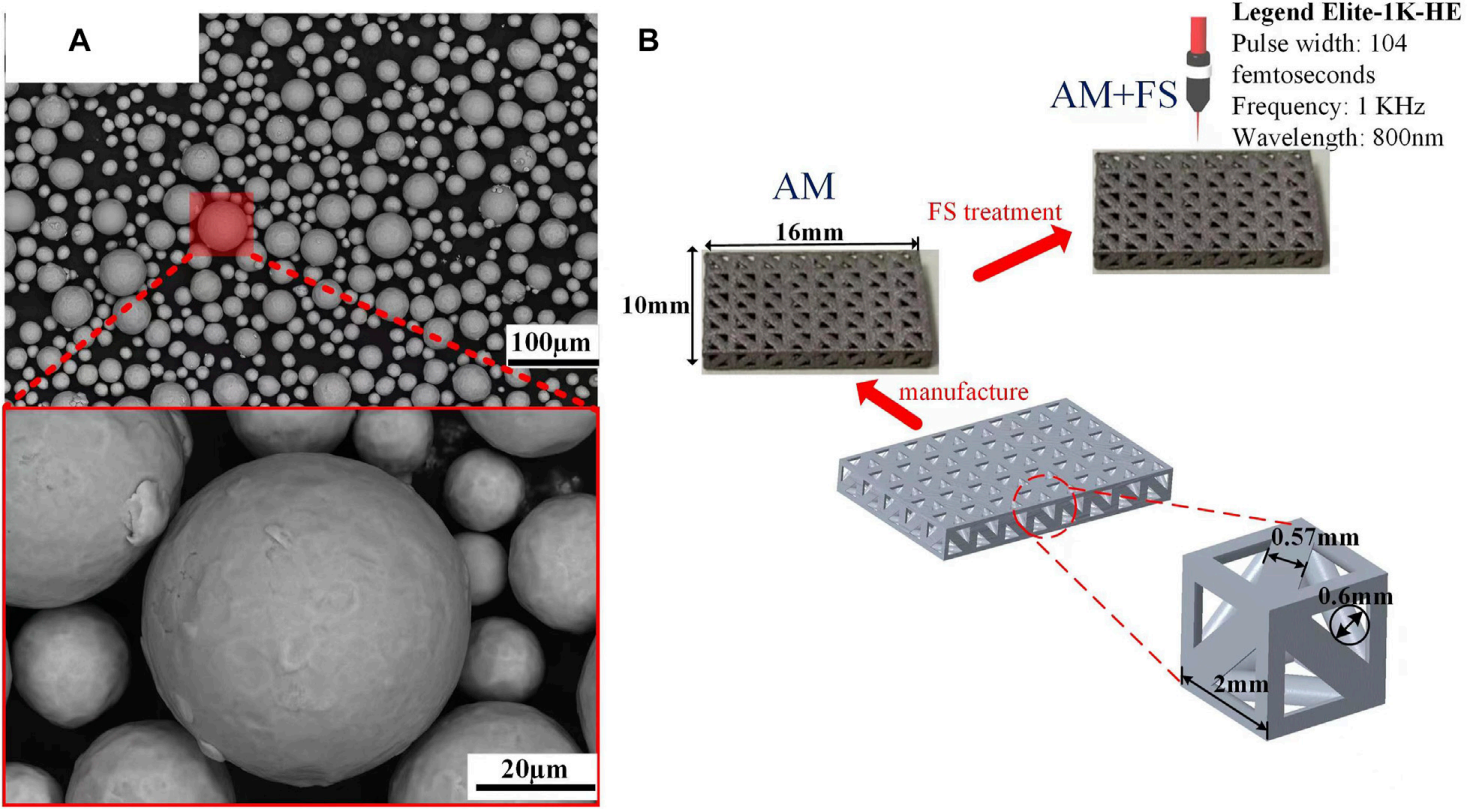
Materials and samples: **(A)** SEM image of the titanium alloy powder; **(B)** bone scaffold sample.

In this study, the particle size of Ti_6_Al_4_V powder (Avimetal Powder Metallurgy Technology (Beijing) Co., Ltd.) ranges from 15 to 53 μm. The degree of sphericity is quite well **(** Figure 1A **)**. The main components of the titanium alloy powder are presented in Table 1. The bone scaffold is generated by a periodic array of pillar tetrahedral basic cells (TBCs) and designed by Unigraphics NX (Siemens PLM Software, Germany). The design method and structural parameter relationship can be found in our previous articles (Wang et al., 2019). The unit porosity is 65 %, and the typical dimension is 16 mm by 10 mm **(** Figure 1B).

**TABLE 1:** Chemical composition of the titanium alloy powder.

Ti	V	Al	C	Fe	O
Bal	3.82	5.83	0.023	0.068	0.12

Commercial SLM tools were used to create the bone scaffolds (FS271M, Sichuan Huashu Turing additive manufacturing technology Co., Ltd., Chengdu, Sichuan). The parameters of this equipment are as follows: the laser power is 500 W (W), the radius of the laser facula is 35 μm, the layer thickness is 30 μm, and the laser scanning speed is 240 mm/s. The scanning technique was designed as 67°- and 125°-angled laser channels for the neighboring layers to melt powder molding in order to eliminate internal residual stress. To ensure that printing was carried out in a pure argon gas environment, oxygen in the forming cavity was removed before printing with argon gas that had a purity of 99.999 %. The bone scaffolds were created in the end **(** Figure 1B). After printing, wire electrical discharge machining (WEDM) technology was used to separate bone scaffold samples, which was created by additive manufacturing (AM), from the substrate. To eliminate contaminants and pollutants, the samples were then ultrasonically cleaned in industrial alcohol for 5 minutes.

The laser beam from the regeneratively amplified titanium gem femtosecond laser system abraded the surfaces of the AM samples with pulse times of 104 femtoseconds, repetition rates of 1 kHz, and center wavelengths of 800 nm (Legend Elite-1K-HE, Coherent, United States). The femtosecond laser surface treatment experiment was carried out at room temperature with air as the medium. During the ablation process, the laser beam guided the AM sample surfaces through the galvanometer scanning system (intelliSCAN III 14, SCANLAB), focusing the laser beam and scanning along with the linear scanning method. The scanning laser power was 50 mW, the speed was 0.5 mm/s, the focus spot was circular, the radius of the laser facula was 25 μm, the chirp of the pulse was 0.5 μm, and the line spacing was 0.04 mm. The new additive manufacturing (AM + FS) samples were taken after the femtosecond laser treatment and immersed in acetone for 5 min of ultrasonication to remove any remaining debris from the surface.

### Surface characteristics of bone scaffolds

The surface morphology of the AM samples and the AM + FS samples was quantitatively analyzed using an atomic force microscope (AFM, NT-MDT, Russia). Five samples from each group were measured for their roughness average (Ra), roughness kurtosis (Rku), and roughness skewness (Rsk), and their averages were computed. Samples were scanned using an AFM at a width of 20 F0B4 20 mm. A field emission scanning electron microscope (SEM, JSE-5900 L V, Japan) was used to examine the surface morphology of the AM and AM + FS samples.

The hydrophilicity of the bone scaffold surface was evaluated by measuring the contact angle of droplets on the sample surfaces, which were detected by an optical contact angle-measuring instrument system (SDC-350, Shengding Precision Instrument Co., Ltd., Dongguan, China). Deionized water was used as the test solution, and a microliter syringe was used to control the droplet size to 2 µL. Contact angle (CA) represents the average value obtained from different plane measurements.

### *In vitro* bioactivity test of bone scaffolds

For 14 days at 37°C, five samples from the AM and AM + FS groups were immersed in a simulated bodily fluid (SBF) to examine the scaffold surface’s capacity to promote HAP formation. The SBF used in the experiment was purchased from Fuzhou Beijing Biotechnology Co., Ltd. The ratio of the SBF solution volume to scaffold mass was 200 ml/g. It was noted that the volume of the SBF solution remained constant during the soaking process to ensure sufficient reaction ions for HAP formation. After soaking, the sample was taken out and repeatedly washed with ethanol three times to prevent further reaction. Then, the HAP deposition on the surface of the bone scaffold was evaluated and confirmed by SEM and energy-dispersive spectroscopy (EDS).

### Rabbit tibial stent implantation experiment

#### *In vivo* implantation of bone scaffolds

This experiment was carried out in the experimental animal center of Southwest Medical University (Ethics No.: 2020878). A tibial metaphysis implantation experiment was conducted on 36 rabbits (weighing about 2.5 and 3 kg) at the age of 3 months.

**FIGURE 2:**
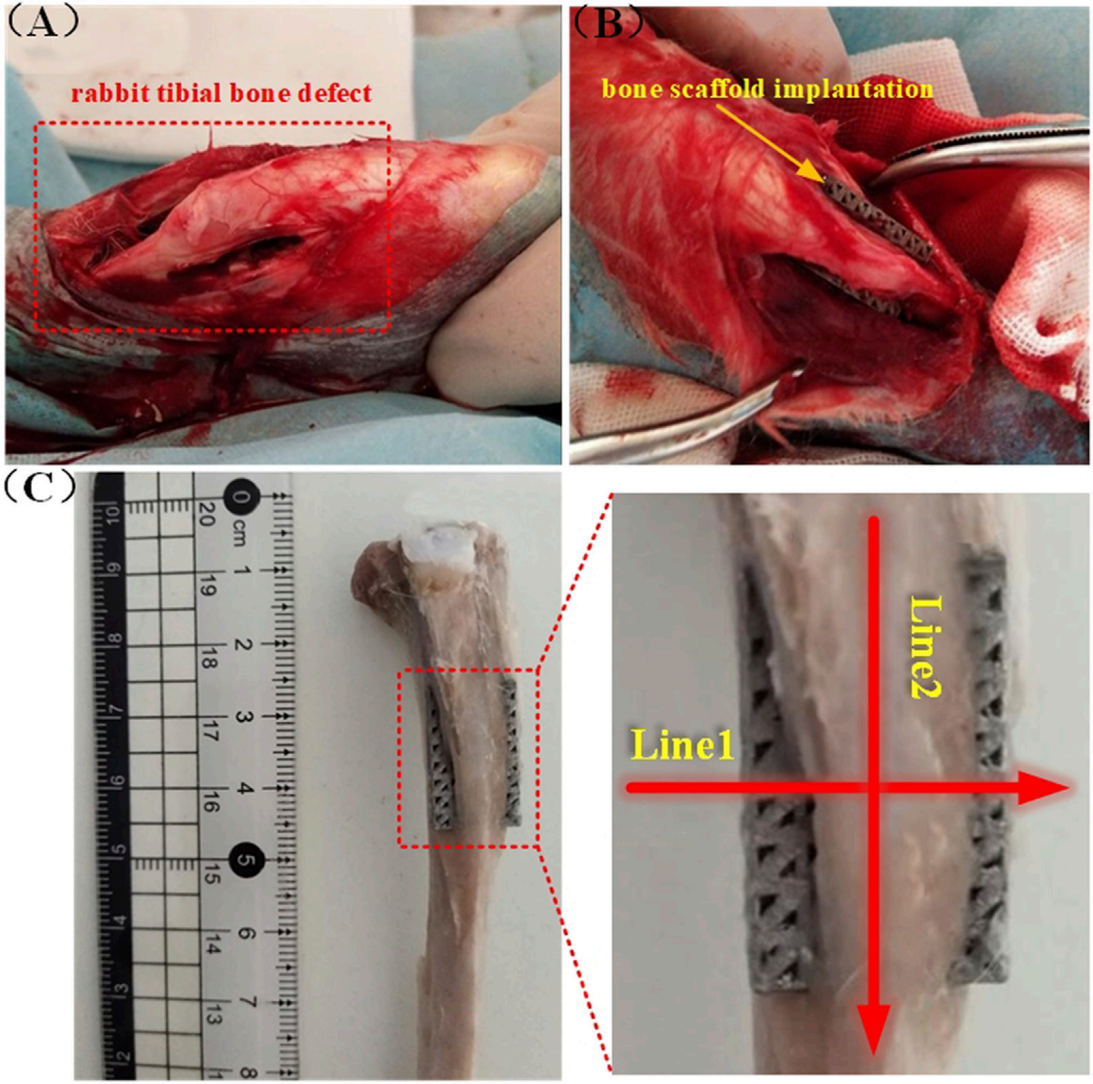
Rabbit tibial implantation experiment: **(A)** Rabbit tibial defect; **(B)** bone scaffold implantation; **(C)** schematic diagram of the cutting line of the hard tissue section after removal of the implanted bone scaffold.

Three groups of 12 rabbits each, made up of 36 rabbits, were randomly selected to represent three time-points (2, 4, and 8 weeks). At each time-point, the right tibial epiphysis of 12 rabbits was randomized to receive AM and AM + FS sample implants. A rectangular defect was drilled with a dental drill at the epiphysis of the tibial shaft after the rabbits were anesthetized with 3 % pentobarbital sodium (30 mg/kg), then a bone scaffold was randomly implanted in the defect area, and the incision was sutured (Figures 2A,B). The rabbits were treated with penicillin for 3 days after the operation and fed separately. The rabbits were killed by intravenous injection of excessive sodium pentobarbital at 2, 4, and 8 weeks after implantation. For further analysis, the bone scaffold around the tibial metaphysis was immersed in 10 % formalin solution.

### Micro-CT experimental evaluation

Micro-CT (SCANCO Medical, Switzerland) scanning was performed on the tibia metaphysis-containing stent at weeks 2, 4, and 8, respectively, and the scanned files were imported into Mimics 21.0 (Materialise, Belgium) software for reconstruction. Micro-CT was used to quantitatively analyze the growth of bone tissue into bone scaffolds. The bone tissue’s interstitial growth capacity was quantified by the ratio of the new bone volume (BV) of the scaffold to the total volume (TV) of the analysis area.

### Histological experimental evaluation

After Micro CT scanning, the specimens were dehydrated with 70 %, 80 %, 90 %, and 100 % ethanol, respectively, and then soaked in a formalin solution at 37°C for 1 week. A hard tissue section machine (SP1600, Leica, Germany) was used to slice the tissue along the radial and axial directions of the tibia. The slice thickness was 50 µm, and the schematic diagram showed the tissue slice line (Figure 2C). The radial and axial directions of the femur were labeled as Line1 and Line2, respectively. After cutting it into sections, van Gieson stain, which contains 1.2 % trinitrophenol and 1 % acid fuchsin, was applied, and the development of bone tissue was seen under an optical microscope (DMCA, Leica, DM2500, Germany).

## Results and Discussion

### Surface characteristics of bone scaffolds

**FIGURE 3:**
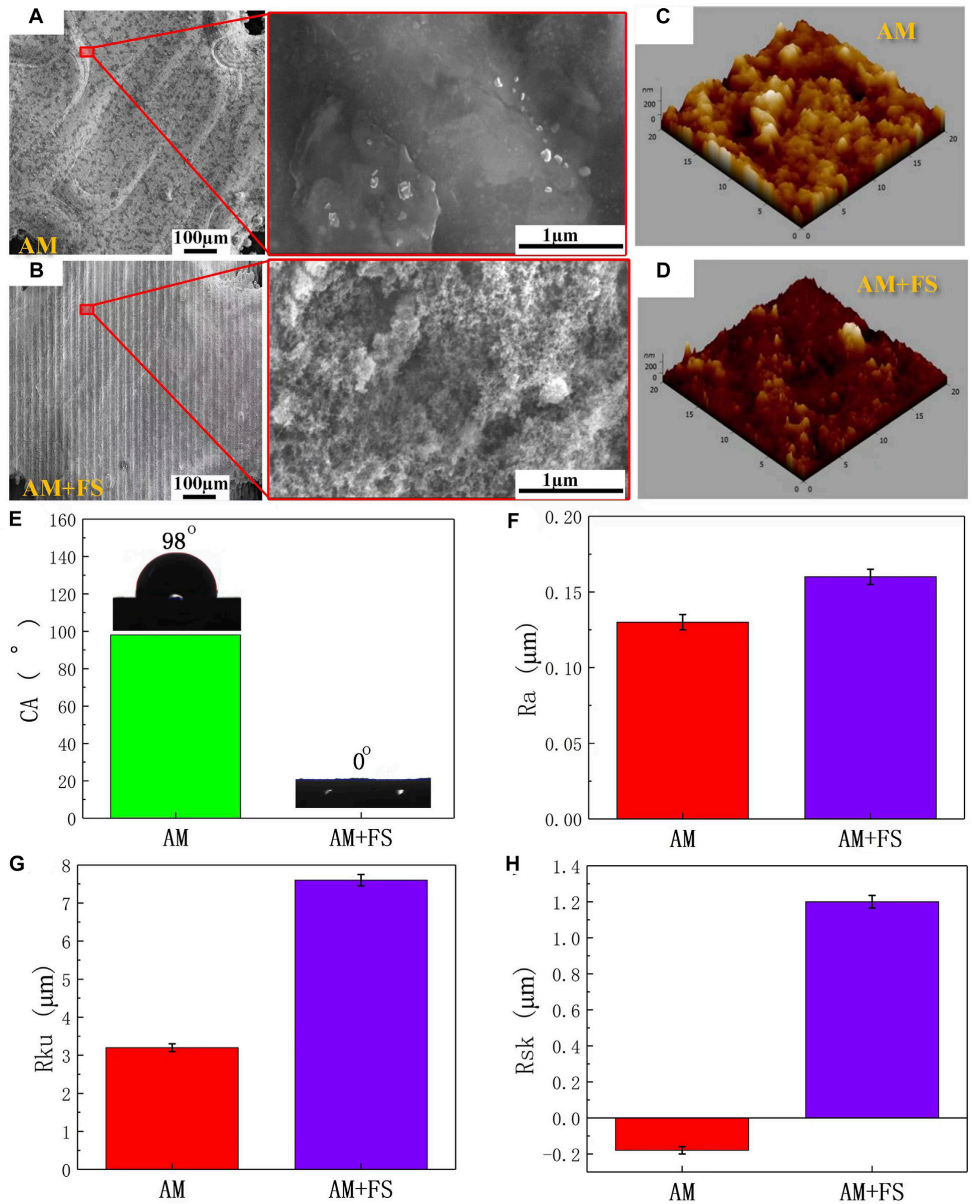
Surface characteristics: **(A–B)** SEM images and optical microscopic images of the surface of AM and AM + FS samples; **(C–D)** AFM three-dimensional morphologies of the AM and AM + FS samples; **(E)** the water contact angles of the sample surface; **(F–H)** surface parameters of Ra, Rku, and Rsk of samples.

Figures 3A, B present the surface morphology of SEM images and optical microscopic images of AM samples and AM + FS samples. It might be seen that the surface morphology of the AM sample was relatively smooth, while that of the AM + FS sample after femtosecond laser surface treatment changes, forming a micro/nano-structure surface with a similar osteoporosis structure. Previous research has demonstrated that a critical feature influencing the bioactivity of metal implants is their surface morphology. Cells respond by detecting the surface morphology properties of implants, leading to adhesion, proliferation, and differentiation (Feller et al., 2014; Li et al., 2016; Liu et al., 2020). In particular, the surface morphology of the micro/nano-level combination improved the surface roughness and hydrophilicity, which was beneficial to the adhesion and differentiation of osteoblasts (Gittens et al., 2014a; Chen et al., 2017; Shaikh et al., 2018).

AFM was utilized to capture the three-dimensional surface morphology of AM and AM + FS samples, and roughness parameters were employed to quantitatively explain the surface morphology difference brought about by the femtosecond laser treatment of bone scaffolds **(** Figures 3C,D)**.** The roughness of the two sets of samples was assessed in this study using the roughness metrics Ra, Rku, and Rsk. Ra represents the unevenness of the surface, and the larger the value, the rougher is the surface; Rku indicates the sharpness of the surface, and the higher its value, the sharper is the peak surface (Rku >3 indicates that the surface is the peak surface); Rsk represents the asymmetry of the surface, where a positive value represents the dominant number of surface peaks, and a negative value represents the dominant number of surface valleys. The results show that the surface roughness parameters of Ra, Rku, and Rsk of the bone scaffold AM + FS samples were higher than those of AM samples, indicating that the surface of the bone scaffold treated by femtosecond laser had higher roughness (Figures 3F, H).

The surface of the bone scaffolds underwent femtosecond laser treatment, which caused the unevenness to worsen and a sharper surface peak to develop. The analysis results of the skewness value demonstrated that although the surfaces of the bone scaffold AM samples and AM + FS samples were peak surfaces (Rku>3), the surface Rsk of the AM samples were negative, and the number of surface valleys was more than that of the surface peaks. After femtosecond laser surface treatment, the surface tended to form surface peaks rather than surface valleys, and the number of surface peaks on the surface of the AM + FS sample was much greater than that of the surface valleys. It is more conducive to cell adhesion and speeds up the proliferation and differentiation of bone tissue cells when the surface area of a bone scaffold in contact with bone tissue cells rises (Chan et al., 2017; Spriano et al., 2018).

Surface hydrophilicity is another crucial factor determining the interaction between the surface of the bone scaffold and surrounding osseous tissue in addition to the surface morphology and roughness (Gittens et al., 2014b; Rupp et al., 2014). The water contact angles were measured to evaluate the hydrophilicity of the sample surfaces (Figure 3E). The average water contact angle on the surface of the AM samples was 98°, which was the hydrophobic structure. The average water contact angle on the surface of the AM + FS samples was almost 0°, which was the super-hydrophilic structure. The effectiveness of HAP deposition, cell adhesion, and bioactivity is significantly influenced by the hydrophilicity of the bone scaffold surface (Chen et al., 2014; Zhang et al., 2019; Menazea and Ahmed, 2020). The adhesion of cells at the initial stage of implantation has a significant impact on the proliferation and differentiation of subsequent cells, and relevant studies have shown that fibronectin and other important extracellular matrices (ECM) related to cell adhesion tend to be adsorbed on the hydrophilic surface (Wilson et al., 2005). As a result, the hydrophilic scaffold surface can bind to osseous tissue *in vivo* more than the hydrophobic scaffold surface.

### *In vitro* bioactivity evaluation of bone scaffolds

**FIGURE 4:**
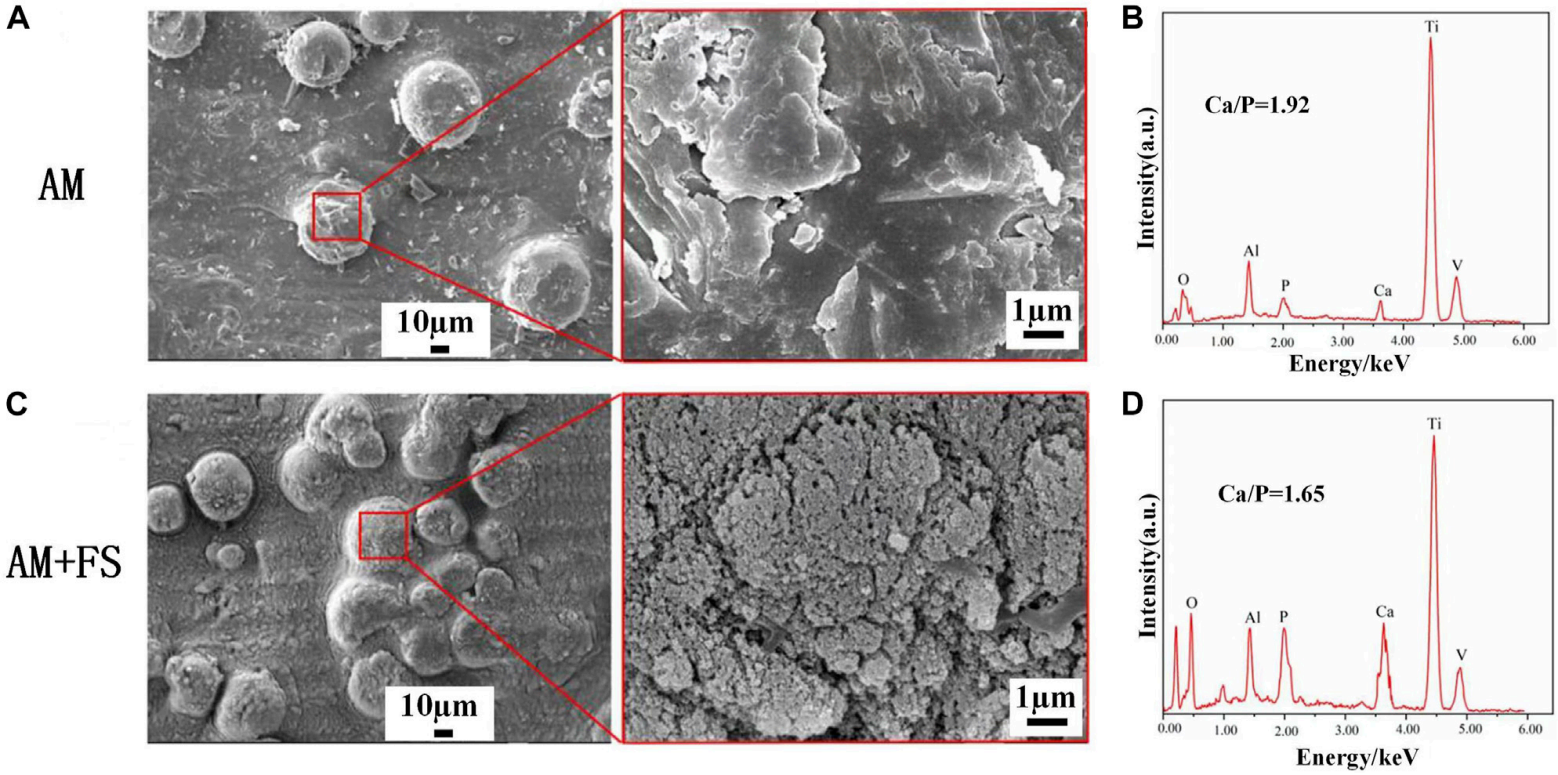
SEM and EDS of the surface of samples soaked in SBF for 14 days: **(A–B)** AM samples; **(C–D)** AM + FS samples.

The formation of HAP is an important sign of the bioactivity of materials. It is also one of the criteria for evaluating the bioactivity of bone repair materials *in vitro*. The AM samples and AM + FS samples were soaked in SBF for 14 days. The surface morphology of the bone scaffolds soaked for 14 days was observed by SEM (Figures 4A–C).

HAP is clustered and composed of many nanocrystals. This is the typical form of HAP deposited in SBF. HAP was observed on the surface of AM samples and AM + FS samples, indicating that the titanium alloy bone scaffold had the ability to induce HAP deposition before and after femtosecond laser surface treatment. It is worth mentioning that only few HAP particles were generated on the surface of the AM samples, and the HAP coverage was low. However, a dense HAP layer was formed on the surface of the AM + FS samples, and the coverage was significantly increased. As a result, the bone scaffolds treated with the femtosecond laser had a stronger ability to induce HAP deposition and better bioactivity.

The elemental composition of the sample surface was analyzed by EDS (Figures 4B–D). The AM + FS sample’s Ca/P ratio was about 1.65 after 14 days of soaking in the SBF solution, comparable to the Ca/P ratio of HAP typically seen in human bones. Compared to the typical Ca/*p* value of HAP in human bones, the AM samples had a higher Ca/*p* ratio of around 1.92. In an *in vitro* SBF immersion experiment, calcium ions were first deposited on the surface, and then, HAP grew there (Zhao and Xiong, 2012). Even while HAP is generated on the surface, it happens significantly more slowly in AM samples than in AM + FS samples, according to the AM sample’s greater Ca/P ratio. The results of the EDS analysis and the SEM observation findings are in agreement.

### Evaluation of the bone ingrowth ability of bone scaffolds *in vivo*

#### Micro-CT evaluation

**FIGURE 5:**
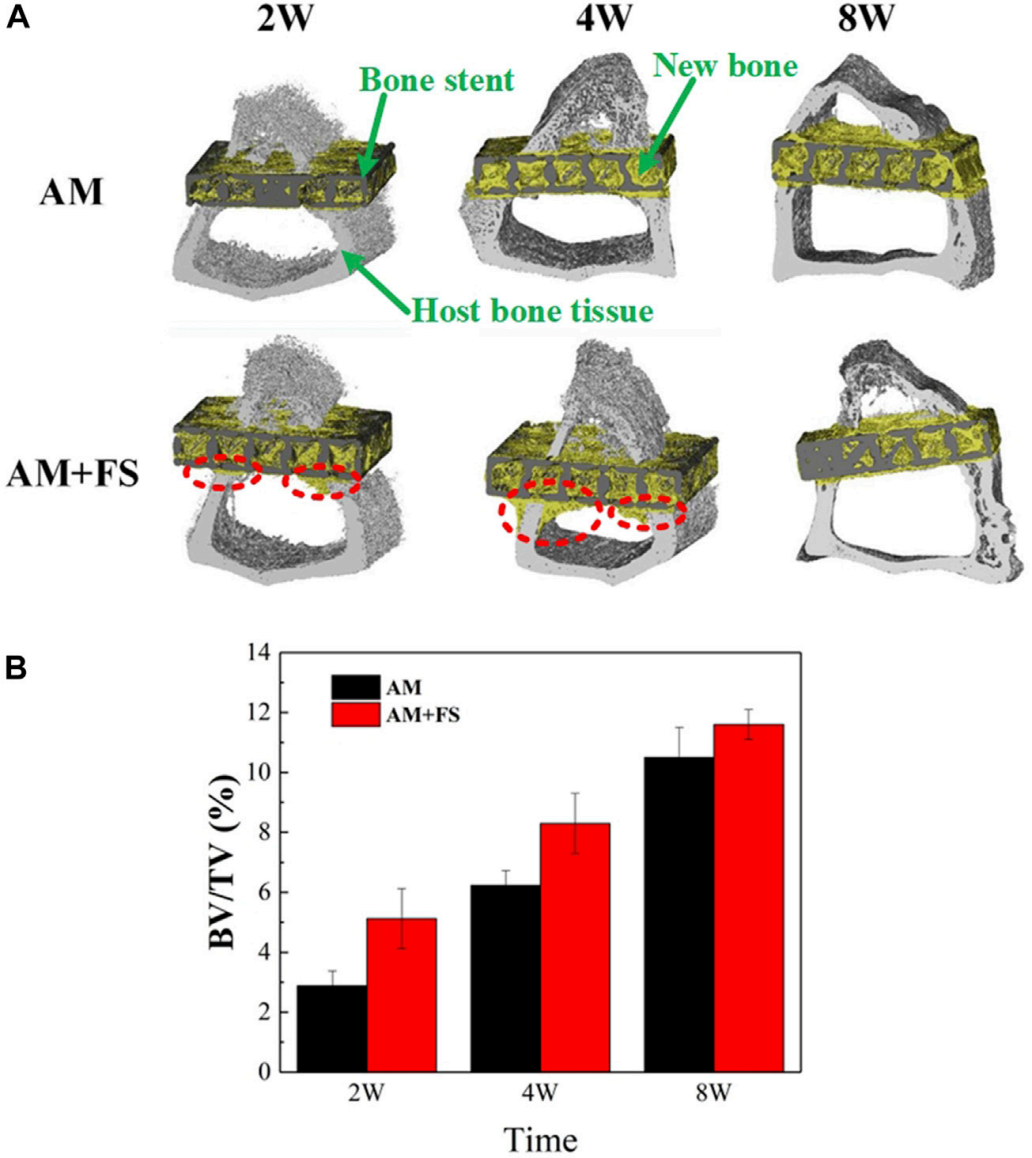
Micro-CT evaluation: **(A)** micro-CT reconstruction model of bone stent implantation in rabbits at different time-points; **(B)** BV/TV values at different time-points of bone scaffold implantation in rabbits (*p < 0.05).

*In vitro* experimental results have shown that femtosecond laser treatment can improve the surface bioactivity of bone scaffolds. To further test the efficiency of osseointegration *in vivo*, the AM and AM + FS samples were implanted into the epiphysis of the rabbit tibial shaft for 2, 4, and 8 weeks, respectively. The ratio of the BV to the TV was used as an index to evaluate the bone ingrowth ability of the bone scaffold. The higher the value of BV/TV, the more the scaffold grows into bone tissue and the better is the osteogenic growth performance of the scaffold. The micro-CT three-dimensional model was reconstructed at different time-points after the bone scaffold was implanted into the rabbit tibia, in which the black part was the titanium alloy scaffold, the yellow part was the new bone tissue, and the gray part was the host’s original bone tissue (Figure 5A). It was noted that due to the length of the bone scaffold implant, only a 5-mm long section was intercepted for analysis during 3D reconstruction. Since there were a certain number of bone tissue cells in the bone marrow cavity, a small amount of bone tissue cells had adhered and proliferated on the surface of the bone scaffold in the early stage of implantation. This indicated that the titanium alloy bone scaffold manufactured by SLM had excellent biocompatibility.

As can be seen from the three-dimensional reconstruction model (Figure 5A), with the increase in implantation time, there was an increasing number of new bone tissues in the scaffold. In the first 2 weeks of implantation, it might be seen that the cortical bone tissue of the AM + FS samples extended to the marrow cavity along the contour of the scaffold, which was not the case in the AM samples. At the fourth week after implantation, the new bone tissue of cortical bone cells in the AM + FS samples increased gradually along the contour of the scaffold. In contrast, the bone tissue in the AM samples did not grow to the bone marrow cavity along the contour of the scaffold, which showed that the surface of the bone scaffold had better biocompatibility and osteoconduction after the femtosecond laser surface treatment. At the eighth week of implantation, it might be seen that many new bone tissues were growing in the AM samples and AM + FS samples. Nevertheless, compared with the AM samples, there were more new bone tissues in the pores of AM + FS samples. Bone tissue and scaffolds have become a whole, forming good osteointegration.

The quantitative analysis results of the bone growth ability after bone scaffold implantation by micro-CT were described by the BV/TV ratio (Figure 5B). It can be seen from the second week of implantation, the new bone volume of the AM + FS sample was almost twice that of the AM sample, indicating that the surface bioactivity and bone ingrowth ability of the bone scaffold were significantly improved after the femtosecond laser surface treatment. In the early stage of bone scaffold implantation, osteoblasts first adhered to the scaffold surface and then gradually grew into the scaffold pores. The surface of the bone scaffold treated by the femtosecond laser provided a good place for cell adhesion in the early stage of growth. At the fourth and eighth weeks after implantation, the volume difference of the new bone between the two groups’ scaffolds decreased gradually, which was mainly attributed to the limited space provided by the 2 mm thickness of the scaffold for bone tissue growth. Therefore, the growth of bone tissue in the AM + FS samples slowed down gradually in the later stage.

### Histological evaluation

The hard-tissue section machine was used to slice the tissue along with the schematic diagram of the slicing line (Figure 2C), stain after slicing, and observe the growth of bone tissue under an optical microscope.

**FIGURE 6:**
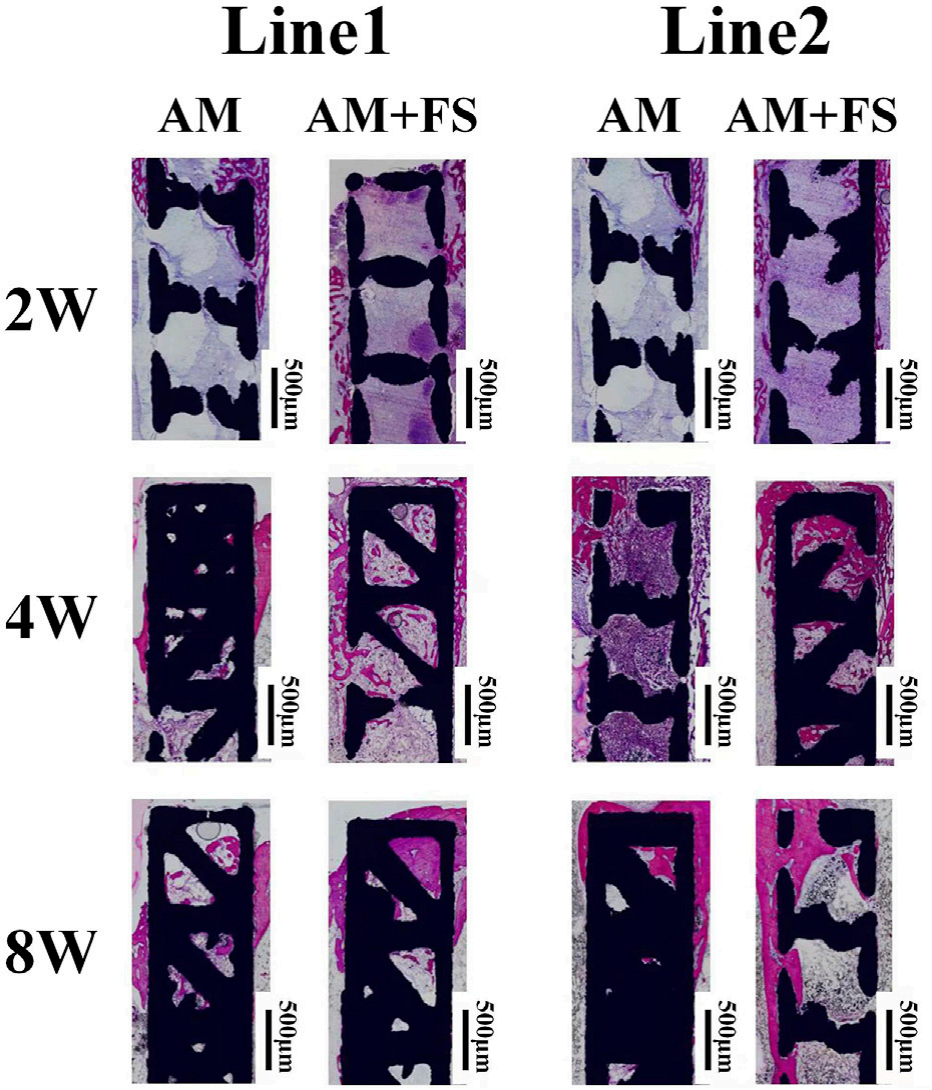
VG stain histological sections of AM and AM + FS samples at different time-points.

After the implantation of bone scaffolds, at weeks 2, 4, and 8, bone tissue development was seen (Figure 6). In the figure, the scaffold is shown in black, the new bone is shown in red, and the fibrous tissue is shown in blue. The radial and axial directions of the femur are shown by lines 1 and 2, respectively.

For the Line1 direction, the bone tissue first grew from the place where the cortical bone contacted the scaffold to both sides (Figure 6). In the second week, the bone tissue cells of the AM + FS samples proliferated bilaterally. However, only a few cells proliferated bilaterally in the AM samples. In the fourth week, the bone tissue cells of the AM + FS samples proliferated bilaterally and expanded into the inner pores of the scaffold. In the AM samples, bone tissue cell growth remained mostly unaltered. There were considerably less bone tissue cells in the inner pores of the AM samples than the AM + FS samples. In the eighth week, bone tissue could be found to be present in the inner pores of the AM samples and the AM + FS samples in contact with the cortical bone. The AM + FS samples had a wider contact area with bone tissue than the AM samples did, and the bone tissue interlocked well with the scaffold.

For the Line2 direction, because the scaffold was not in direct contact with the cortical bone, the bone tissue cells on the scaffold proliferated from the contact part between the cortical bone and scaffold. The capacity of bone tissue cells on the scaffold to proliferate and differentiate is directly reflected in the direction in which the bone tissue is growing. In the second week of the AM + FS samples, it can be seen from the Line2 direction that a few bone tissue cells proliferated to the center of the scaffold **(** Figure 6). At the same time, for the AM samples, only a few bone tissue units proliferated at the edge of the cortical bone. At the fourth week, there was only a small amount of bone tissue cells in the middle of the AM samples, while more bone tissue cells had adhered to the middle of the AM + FS samples and proliferated and differentiated in the pores of the scaffold. At the eighth week, bone tissue cells had proliferated and differentiated well along the lateral side of the AM + FS samples, and the degree of cell proliferation on the AM samples was significantly lower than that of the AM + FS samples.

In conclusion, the *in vivo* implantation experiment showed that AM + FS samples in the Line1 direction might see the adhesion and growth of bone tissue cells on the scaffold surface after 2 weeks of implantation, showing good osseointegration ability. Compared with the AM samples at the same time point, the AM + FS samples showed more new bone tissue growth, indicating that the surface after femtosecond laser treatment can promote the adhesion of bone tissue cells. More bone tissue in the Line2 direction showed proliferation along the surface of the AM + FS samples, and bone tissue and bone scaffolds showed good mechanical interlocking characteristics at the eighth week of implantation.

## Conclusion

In this study, the surface of Ti alloy bone scaffolds made by SLM was treated with a femtosecond laser, and the changes in the surface morphology, roughness, and hydrophilicity of the bone scaffolds following treatment were noted. The bioactivity of the AM and AM + FS samples was measured using the simulated bodily fluid immersion technique. To assess the bone development performance *in vivo*, the bone scaffolds were transplanted into the metaphysis of the rabbit tibia. The most innovative aspect of this study is how thoroughly and methodically the aforementioned tests have been conducted and studied. The key findings are as follows:

1) Bone scaffolds that had been surface-treated with a femtosecond laser developed micro–nano surface topography, which increased surface roughness and hydrophilicity.
2) *In vitro* activity tests revealed that treating the surface of the bone scaffold with a femtosecond laser resulted in dense HAP, which had improved bioactivity and promoted the adhesion and proliferation of bone tissue cells.
3) The implantation of a bone scaffold in the rabbit tibia metaphysis resulted in more new bone tissues growing in the AM + FS samples than in AM samples alone. It was further established that treating the bone scaffold with a femtosecond laser improves bone tissue cell adhesion and proliferation. Additionally, the growth of bone tissue on the surface of the AM + FS samples demonstrated strong mechanical interlocking properties with bone scaffolds.

## Data Availability

The original contributions presented in the study are included in the article/Supplementary Materials; further inquiries can be directed to the corresponding authors.
